# ‘South Asian cocktail’ - the concurrent use of opioids, benzodiazepines and antihistamines among injecting drug users in Nepal and associations with HIV risk behaviour

**DOI:** 10.1186/1477-7517-11-17

**Published:** 2014-05-23

**Authors:** Saroj Prasad Ojha, Suraj Sigdel, Hans-Günter Meyer-Thompson, Harald Oechsler, Uwe Verthein

**Affiliations:** 1 Tribhuvan University Teaching Hospital (TUTH), Maharajgunj, GPO Box 3578, Kathmandu, Nepal; 2 Mental Health and Participatory Research Center, GPO Box 20938, Kathmandu, Nepal; 3 Department of Psychiatry and Mental Health, Substance Abuse, ASKLEPIOS Clinics North, Hamburg 22419, Germany; 4 Centre for Interdisciplinary Addiction Research (CIAR), Hamburg University, Hamburg 20246, Germany

**Keywords:** Opiates, Benzodiazepines, Antihistamines, South Asian cocktail, Opioid substitution treatment, HIV, Buprenorphine (injectable)

## Abstract

**Background:**

Data of the Central Bureau of Statistic of Nepal from 2008 show a total of more than 46,000 illegal drug users, out of which 61% are injecting drug users (IDU). An injecting mix of medicines like opioids, benzodiazepines and antihistamines (the so-called South Asian cocktail) was prevalent. Furthermore, it is estimated that about 70,000 people are living with human immunodeficiency virus (HIV). The government of Nepal has started realizing and recognizing drug use and HIV as significant health and social issues. Harm reduction programs such as needle syringe exchange and opioid substitution treatment are being implemented.

**Methods:**

The aim of this study is to obtain specific knowledge on the drug use behaviour and the health status of drug users with a focus on HIV in drug users with concurrent injection of opioids, benzodiazepines and antihistamines. After an initial mapping of Kathmandu Valley, 300 drug users in contact with different treatment and counselling centres were randomly chosen for the interviews. The research questionnaire was designed according to the European Addiction Severity Index (EuropASI) and Maudsley Addiction Profile standards.

**Results:**

Ninety-one percent of the respondents are male and 9% female. Mean age is 28.7 years. Ninety-five percent are injecting drug users with a mean of 8.7 years of drug use history. Eighty-six percent are injecting different ‘cocktails’, usually made of buprenorphine, diazepam, promethazine and/or other substances (30-day prevalence). Similarly, 48% use heroin, whereas only 2% take cocaine/crack. Among those tested for HIV (*N* = 223), 33% are positive (25% of the sample population). Compared to the other drug users (mainly heroin), the cocktail users show a higher HIV infection rate and more co-infections. Furthermore, risk behaviour, as e.g. needle sharing, is much more common among the cocktail users.

**Conclusion:**

Currently, the mixture of medicines, opioids, benzodiazepines and antihistamines, is the predominant drug in Nepal; the pharmaceutical drugs needed to prepare the cocktail are less expensive than heroin and relatively easy to acquire. The cocktail users show a higher risk behaviour regarding the transmission of HIV than heroin drug users. It needs to be considered which HIV prevention measures are necessary to target the specific needs of drug users who inject a mixture of opioids, benzodiazepines and antihistamines, since the available services (such as needle syringe exchange) do not seem to cover their specific needs (high percentage of needle sharing).

## Introduction

People who use drugs are at risk of getting infected with human immunodeficiency virus (HIV) and of transmitting the infection to other drug users via needle-syringe sharing or to their sex partners through unsafe sex. Injecting drug users can act as a bridge to transmit HIV to others with whom they have sexual contacts. According to the Integrated Biological and Behavioral Surveillance (IBBS) report 2011, 76% of injecting drug users (IDUs) in Kathmandu Valley consistently used condoms with female sex workers, while 40% used condoms consistently with non-regular sex partners and only 9% used condoms consistently with regular female sex partners in the past year [1]. Numerous studies have found IDUs to be disproportionately likely to be involved in unsafe sex practices [2,3]. Sex workers (male and female), who offer sex in exchange for money or drugs, are at high risk for HIV infection and can spread the virus to a large number of people. In addition, sexual contact between IDUs and non-injectors may also contribute to an increased incidence of HIV infection. Furthermore, HIV transmission to children by drug-injecting mothers (‘vertical transmission’) or by non-injecting mothers in partnership with IDUs is pervasive. As a result of high-risk sexual behaviour, HIV is also a risk among drug abusers who do not inject drugs.

Until the 1990s, injecting drug use was rare in Nepal. With the introduction of pharmaceutical drugs such as buprenorphine, benzodiazepines, chlorpromazine and dextropropoxyphene, a transition took place, away from smoking or chasing heroin and towards injecting drug mixtures, mainly of buprenorphine mixed with benzodiazepines and chlorpromazine, as well as other antihistamines—the so-called South Asian cocktail. Polydrug use appeared to be the norm (ranging from alcohol to heroin), and transition from non-injecting to injecting behaviour appeared to be linked to the need for choosing the most cost-effective route of administration [4].

As per the study conducted by the Central Bureau of Statistic (CBS) of Nepal, more than 46,310 people are using illicit, ‘hard’ drugs such as opiates, cocaine or amphetamines in Nepal [5]. Most of them are male (92.8%); only 7.2% are women. A major share of this number, i.e. 61.4%, is constituted by injecting drug users. It is estimated that in Nepal, 70,256 people are living with HIV [6]. A substantial proportion of IDUs are HIV-infected. However, in the metropolis region of Kathmandu, the prevalence of HIV infections among IDUs has decreased from 51.7% in 2005 to 20.7% in 2009 [7]. According to the IBBS 2011 report, HIV prevalence was found in 16.3% of IDUs who had been injecting drugs for more than 5 years, while no HIV infections were found among those who had injected for less than 1 year [1].

The impact of many types of psychoactive substances, including alcohol, and regardless of the fact whether they are injected or not is risky to the extent that they are dis-inhibitors and affect the individual's ability to make decisions about safe sexual behaviour. In this context, the non-governmental organization ‘Recovering Nepal’ has found, according to its study from 2009, that IDUs are using multiple drugs as a mixture of injectable substances in order to achieve a stronger effect [8]. This is also supported by the IBBS 2011 report which states that IDUs in Kathmandu Valley mostly used a combination of drugs. In fact, about 89% of the IDUs used different combinations of drugs [1]. According to Larance and colleagues, buprenorphine has reportedly emerged as the favoured drug of injection among IDUs [9]. Also, in India, an increase in the consumption of prescription opioids such as buprenorphine, codeine and dextropropoxyphene among drug users has been observed in the past decade [10]. This so-called cocktail use behaviour has created health, social, economic and legal hazards to IDUs.

The coverage of opioid substitution treatment (OST) in Nepal is comparatively low, and high-risk injecting and sexual behaviour among IDUs continues. The financing of OST is largely provided by external donors, and donations have become scarce with the current global economic problems [11].

This study was conducted in order to understand the current situation of illegal drug use with a special focus on concurrent injection of a mixture of opioids, benzodiazepines and antihistamines in Nepal and to gain knowledge on the use of such ‘drug cocktails’ and its hazards in IDUs. In particular, this study aimed at identifying possible differences between drug users with and without concurrent consumption of opioids, benzodiazepines and/or antihistamines with respect to their living situation, health status, drug use history, different types of combinations of drugs, risk behaviour, causes and motives of consumption as well as the association of drug use and risk behaviour with HIV status.

## Methods

### Subjects

A cross-sectional survey among opiate users in contact with the treatment system was conducted. As per the CBS study [5], in 2008, around 17,458 drug users resided in the Kathmandu Valley (Kathmandu, Lalitpur and Bhaktapur). As the research focuses especially on concurrent use of opioids, benzodiazepines and antihistamines, Kathmandu Valley was selected for this survey due to its high concentration of illegal and opiate drug users, including injecting drug users. Out of the 17,000 plus opiate drug users in Kathmandu Valley, 300 IDUs were selected for this study.

Beforehand, a mapping of the institutions/services and the number of patients in treatment in Kathmandu Valley had been carried out in order to calculate the sample size. The sample should consist of about 20% of the target group, but not more than 300 persons.

Patients were randomly chosen from the lists of the respective treatment facilities. If a person refused to take part in the interview the next patient on the list was asked to participate. Altogether 300 drug users (IDUs) were interviewed for this study. The sample size was determined by the expedience method, thereby including those items readily available or convenient to collect.

As the research focuses on illegal drug users, only persons in contact with rehabilitation centres, treatment centres, OST clinics and counselling services were included in the study. Centres from Kathmandu, Lalitpur and Bhaktapur were visited to collect information.

### Face-to-face interviews

Six trained interviewers conducted structured interviews with the participants. After piloting with five respondents, only slight changes were made to the questionnaire. Prior to the study, the interviewers received special training on the content of the interview, the organization of the centres and how to communicate with the drug users.

The interviewers used a questionnaire designed according to standards of the European Addiction Severity Index (EuropASI) [12] and the Maudsley Addiction Profile (MAP) [13] in order to provide comparability with other international studies.

The interviewers requested information on patients' socioeconomic characteristics (age, gender, years of education, marital status and job status), addiction history prior to treatment entry: all drugs used for at least 1 month in their lives, routes of administration, the main drug of abuse (the most problematic drug, i.e. main cause for treatment entry), age at first drug abuse, the duration of drug use and the total amount of money spent on drugs in the most recent month of abuse. In addition, the interviewers also inquired about the patients' ‘cocktail’ drug use history: types of combinations and medicines, history, causes of cocktail use, routes of administration, sources of syringes, cost of drugs, etc.

The interviewers conducted the interviews in cooperation with the staff of the centres who encouraged the selected patients to participate. Patients usually spent some time in the waiting room before visiting the counsellor, receiving their medication or visiting their doctor, thus providing a good opportunity for the interview. The interviewers explained the study to the drug users individually and inquired about their willingness to participate. The data collection in each facility or clinic was continued in order to reach all patients that visited the institution within 1 month.

### Ethical approval

The data collection was carried out using an anonymous patient characteristic form which aimed at providing as much confidentiality as possible. The study was voluntary, and all respondents provided their written informed consent. The National Health Research Council of Nepal approved the study.

### Statistical analysis

With respect to the consumption of drug mixtures, the study participants were divided into three groups. The category ‘intensive use’ is used for persons who took at least two different kinds of cocktails and at least one of them on a daily basis. The ‘moderate users’ consumed the combination of opioids, benzodiazepines and/or antihistamines on at least 1 day (or more) in the past month without fulfilling the above-mentioned criteria. The ‘non-cocktail users’ did not use these drug combinations during the past 30 days.

The data were transferred from the paper forms to a digital format. Statistical data analysis was performed using the Statistical Package for the Social Sciences (SPSS for Windows, version 19 [14]), and any differences in frequencies were analysed using the chi-square test; the ANOVA and *t* test served to compare the means (level of significance *p* < .05).

## Results

### Sample characteristics

Ninety-one percent of the respondents are male and 9% female. Mean age is 28.7 years (range 17 to 51 years); more than half of the drug users are between 26 and 35 years old. The majority is currently in in- or outpatient rehabilitation (together 43%); about one fifth is treated with methadone or buprenorphine. One tenth of the drug users are in contact with drop-in centres. Before entering treatment, almost all respondents had a stable living situation (98%); only 2% lived in hotels or in institutions or were homeless. Two thirds lived in a stable relationship, whereas almost half of this group (in total 32%) had partners with addiction problems of their own. Almost one third reached high school level; 43% finished secondary school. The majority of drug users were working or in training before entering treatment; only one third was unemployed. Two thirds of the sample have been charged for criminal offences at least once in their life (68%); 87% have been incarcerated or imprisoned. During the past 30 days before treatment, 62% have been involved in criminal activities.

### Health status

Forty-seven percent of the Nepali drug users have been hospitalized for medical problems at least once, and 39% suffer from chronic health problems. Among tested drug users (*N* = 223), 33% are HIV positive (25% of the whole sample). Fifty-nine percent of the study participants tested for hepatitis C (*N* = 149) are infected (29% of the whole sample). Eighty-three percent of HIV-positive drug users have co-infections of hepatitis C. In the past 12 months before entering treatment, 43% suffered from abscesses or skin infections.

Among male drug users, 36% are HIV positive; in women, this is true for only one person (4.5%). The mean score of physical health problems according to MAP (range 0–4) is 1.4 for the HIV-positive respondents and 1.3 for the persons without HIV infection (*t* = 1.18, *p* = .238). The MAP mental health score is significantly higher among respondents with HIV infection (2.2) than among those without (1.8) (*t* = 4.5, *p* < .001). Almost one quarter has attempted suicide at least once before in their life (23%), and one third has experienced drug overdoses in their life before entering treatment.

### Drug use history

The majority (95%) are injecting drug users with a mean of 8.7 years of drug use history. Eighty-six percent of the sample were injecting different mixtures of drugs usually made of buprenorphine, diazepam, promethazine and/or other substances (30-day prevalence before entering treatment). Almost half of the sample used heroin (48%), whereas only 2% took cocaine or crack. Cannabis is the most frequently consumed illegal drug among the drug users of Kathmandu Valley. Risk behaviour is very common, i.e. 53% have shared needles and/or syringes, and two thirds have shared other equipment for intravenous use in the past 12 months before entering treatment. According to the number of different kinds of drug mixtures and the frequency of use, 52% are intensive consumers of the so-called South Asian cocktail. Thirty-four percent take those mixtures of drugs on a moderate (less frequent) basis (see below). Fourteen percent of the drug users did not consume opioids in combination with benzodiazepines and antihistamines and/or other drugs in the last month.

Differences between sample characteristics regarding the intensity of concurrent drug use are shown in Table 1. First of all, a significantly higher proportion of men can be found among the users of drug mixtures. The intensive cocktail users are significantly older than the moderate users and have an overall lower level of education compared to the persons without concurrent use of opioids, benzodiazepines and antihistamines and/or other drugs. Intravenous drug use, at least once in life, was found in all cocktail users, i.e. 100% compared to two thirds among the other group. There is also a significantly higher proportion of intensive and moderate drug users who have serious health problems in comparison to the persons without concurrent use of opioids, benzodiazepines and antihistamines and/or other drugs. With respect to co-infections, the prevalence is significantly higher among intensive cocktail users, compared to both other groups of drug users. Similarly, the prevalence of mental health problems is also significantly higher among intensive users of mixed drugs.

**Table 1:** **Sample characteristics and drug use history of the drug users in Kathmandu Valley (*N*** **= 300)**

	**No cocktail use**	**Moderate use**	**Intensive use**	**Significance**^**a **^**(*χ***^**2**^**, ANOVA)**
Gender	74.4% x, y	91.2% x	94.8% y	χ^2^ = 16.6, *p* < .001
Age, years	28.1 (±6.7)	27.6 (±6.2) z	29.6 (±6.1) z	*F* = 3.5, *p* < .05
Currently in OST	18.6%	20.6%	21.3%	*χ*^2^ = 0.1, *p* = .928
Education level				
Primary school^b^	18.6% y	23.5%	32.9% y	*χ*^2^ = 12.4, *p* < .05
Secondary school	32.6% y	48.0%	43.2% y	
High school	48.8% y	28.4%	23.9% y	
Stable living situation	100.0%	96.1%	98.7%	*χ*^2^ = 3.2, *p* = .202
Employment				
Working/student	60.5%	56.9%	57.4%	*χ*^2^ = 2.8, *p* = .593
Unemployed	25.6%	36.3%	32.9%	
Household/other	14.0%	6.9%	9.7%	
Ever injected drugs	67.4% x, y	100.0% x	100.0% y	*χ*^2^ = 87.8, *p* < .001
Length of injecting drug use, years	8.5 (±6.6)	8.4 (±5.5)	10.1 (±6.1)	*F* = 2.9, *p* = .057
Length of cocktail use, years^c^	7.8 (±6.5)	8.0 (±5.0)	9.2 (±5.6)	*F* = 1.7, *p* = .191
Length of heroin use, years	9.9 (±6.0)	11.0 (±6.3)	12.2 (±6.2)	*F* = 2.4, *p* = .090
Serious health problems	4.7% x, y	20.6% x	23.2% y	*χ*^2^ = 7.4, *p* < .05
HIV positive (among tested)	26.9%	24.2%	38.5%	*χ*^2^ = 4.5, *p* = .108
HCV positive (among tested)	38.9%	58.3%	63.9%	*χ*^2^ = 3.8, *p* = .147
Co-infection	18.6% y	31.4% z	47.7% y, z	*χ*^2^ = 15.0, *p* < .001
Physical health (MAP score, 0–4)	1.4 (±0.4)	1.4 (±0.4)	1.3 (±0.5)	*F* = 1.5, *p* = .217
Mental health (MAP score, 0–4)	1.7 (±0.7)	1.7 (±0.7) z	2.0 (±0.8) z	*F* = 4.6, *p* < .05
*N*	43	102	155	

^a^The characters x, y and z indicate significant differences between groups according to *post hoc* statistical tests. ^b^Primary school or lower. ^c^In the group without cocktail use, the number of years is related to former periods of concurrent use of opioids, benzodiazepines and antihistamines and/or other drugs.

### Different types of combinations of drugs

As mentioned above, 86% of the sample population use opioids in combination with benzodiazepines and antihistamines or other substances, i.e. they use it to mix specific cocktails. Forty-five percent of the drug users took three or four different cocktails in the past 30 days. According to the results of the interviews and respondents' statements, the following different types of substance mixtures are consumed by the drug users in Kathmandu Valley. Only 12% of the men and 39% of the women did not use mixed drugs in the past 30 days before the interview (Table 2). On the other hand, it can be shown that different types of substance combinations are used by the same person. The mean number of different cocktails used is 2.0 (±1.3). Based on the persons with concurrent use of different drugs only, the mean number is 2.4 (±1.0). Except for five (male) persons who took cocktail 4 (‘other’) orally, all kinds of cocktails were consumed intravenously.

**Table 2:** **Different types of polydrug injection on a single occasion of use consumed by drug users (*N*** **= 300)**

**Type of drug combination**^**a**^	**Men**	**Women**	**Total**	**Significance (*χ***^**2**^**, *t *** **test)**
1. Buprenorphine + diazepam	64.7%	53.6%	63.7%	*χ*^2^ = 3.2, *p* = .243
2. Buprenorphine + diazepam + promethazine	59.2%	46.4%	58.0%	*χ*^2^ = 1.7, *p* = .193
3. Buprenorphine + diazepam + promethazine + other antihistamine	69.5%	42.9%	67.0%	*χ*^2^ = 8.1, *p* < .01
4. Other (buprenorphine + promethazine or cocktail + pheniramine maleate)	15.8%	7.1%	15.0%	*χ*^2^ = 1.5, *p* = .221
No cocktail use	11.8%	39.3%	14.3%	*χ*^2^ = 15.7, *p* < .001
Mean number of cocktails (±SD)	2.1 (±1.2)	1.5 (±1.5)	2.0 (±1.3)	*t* = 2.0, *p* < .05

^a^The first three kinds of drug mixtures were consumed intravenously only; cocktail number 4 (other) was taken orally by five (male) persons.

### Concurrent drug use and risk behaviour

Different kinds of cocktails are usually consumed several times a day. The majority of intensive users normally take (any kind of) drug mixture three or more times a day (70%). This is also true for the moderate users (69%). Another quarter of both groups consume opioids in combination with different other drugs twice on a typical day (intensive users 26%; moderate users 25%).

As already mentioned above, 52% of the drug users are intensive consumers of combinations of drugs. Empirically, the mean number of different cocktails consumed in this group is 3.0 (±0.6). Thirty-four percent take opioids in combination with other drugs on a moderate (less frequent) basis. Their mean number of cocktails used during the past 30 days is 1.5 (±0.8).

Data on drug use and risk behaviour during the past 30 days was collected from the respondents. Table 3 gives us a picture of the situation. As expected, the percentage of heroin users is the highest in the group without concurrent use of opioids, benzodiazepines and antihistamines and/or other drugs. However, heroin use among intensive cocktail users is significantly higher than in moderate users. Cannabis is more commonly consumed among the groups with combined drug use, whereas only the difference between non-cocktail users and moderate users reached statistical significance. Table 3 reveals that chewing tobacco is also a highly used drug among the intensive users of mixed drugs. Moderate and intensive users also consume a broader range of substances than the group without concurrent use of opioids and/or other drugs.

**Table 3:** **Drug use** (**past 30 days**) **and risk behaviour of the drug users in Kathmandu Valley (*N*** **= 300)**

	**No cocktail use**	**Moderate use**	**Intensive use**	**Significance**^**a**^** (*χ***^**2**^**, ANOVA)**
Alcohol	66.7%	66.7%	60.6%	*χ*^2^ = 1.2, *p* = .558
Number of days	19.1 (±11.7)	22.1 (±10.0)	18.6 (±10.4)	*F* = 2.4, *p* = .098
Heroin	81.4% x, y	29.4% x, z	51.0% y, z	*χ*^2^ = 33.9, *p* < .001
Number of days	27.0 (±7.4) x, y	13.8 (±10.6) x	16.0 (±10.2) y	*F* = 19.5, *p* < .001
Cannabis	69.8% x	87.3% x	80.0%	*χ*^2^ = 6.2, *p* < .05
Number of days	24.8 (±9.1)	21.7 (±9.6)	24.0 (±8.1)	*F* = 2.5, *p* = .088
Benzodiazepines	27.9% x, y	94.1% x	89.7% y	*χ*^2^ = 103.1, *p* < .001
Number of days	22.6 (±9.4)	25.0 (±8.8)	26.2 (±6.8)	*F* = 1.7, *p* = .193
Chewing tobacco	25.6% y	39.2% z	52.3% y, z	*χ*^2^ = 11.2, *p* < .01
Number of days	23.5 (±9.1)	24.5 (±9.1)	21.3 (±9.9)	*F* = 1.5, *p* = .229
Number of different substances	4.7 (±1.8) x, y	6.0 (±1.5) x	6.5 (±1.9) y	*F* = 17.7, *p* < .001
Use of 10-ml syringes	11.8% x, y	61.8% x	56.9% y	*χ*^2^ = 14.9, *p* < .001
Multiple use of the same needle	45.0% x, y	69.6% x	74.8 y	*χ*^2^ = 13.3, *p* < .01
Needle/equipment sharing	48.8% y	61.8% z	90.3% y, z	*χ*^2^ = 43.8, *p* < .001
*N*	43	102	155	

^a^The characters x, y and z indicate significant differences between groups according to *post hoc* statistical tests.

With respect to risk behaviour, the study shows that because needle sharing is significantly more common among intensive cocktail users, they are at a higher risk than moderate users and non-users. Similarly, intensive users also share other equipment very frequently. In both groups of users of mixed drugs, higher percentages of persons than in the non-user group use the same needle several times. In summary, it can be stated that especially intensive users of opioids in combination with other drugs practice risk behaviours significantly more often than the remaining groups.

Most of the intensive cocktail users feel that the concurrent use of these substances alleviates mental symptoms such as depressive feelings or anxiety (59%; moderate users 40%). Much more intensive cocktail users (45%) than moderate users (25%) consume opioids in combination with other substances because the drug (mixture) helps to handle their situation or to ease personal problems. Considering the cost factor, a higher percentage of intensive cocktail users (28%; moderate users 18%) stated to use a mixture of opioids, benzodiazepines and/or antihistamines because they are cheaper than other drugs.

For the intake of these drug cocktails, all drug users use syringes. Most of the drug users generally obtain syringes from pharmacies or drug stores (intensive users 98%; moderate users 100%). A lower proportion of the respondents receive syringes from needle exchange programs in different parts of Kathmandu (intensive users 70%; moderate users 75%). The substances contained in the cocktails are obtained from different sources. The main sources of these substances are drug dealers (intensive users 94%; moderate users 89%) and pharmacies (intensive users 72%; moderate users 80%).

### Drug use and risk behaviour by HIV status

Differences in drug use and risk behaviour become apparent when persons with and without HIV infection are compared (Table 4). The HIV-positive drug users are about 5 years older on average and, correspondingly, have a longer ‘addiction career’ than the HIV-negative persons. Although not statistically significant, there is a tendency for combined use of opioids, benzodiazepines and/or antihistamines to be more prevalent in HIV-positive persons (see also Table 1). Furthermore, a strong association between HIV status and risk behaviour can be shown.

**Table 4:** **Drug use history and risk behaviour of the interviewed drug users in Kathmandu Valley (*N*** = **223**)

	**HIV positive**	**HIV negative**	**Significance (*χ***^**2**^**, *t *** **test)**
Age, years	33.1 (±6.0)	28.2 (±5.6)	*t* = 6.0, *p* < .001
Length of injecting drug use, years	14.2 (±5.7)	8.5 (±5.3)	*t* = 7.3, *p* < .001
Length of cocktail use, years	12.8 (±5.6)	7.8 (±4.5)	*t* = 6.9, *p* < .001
Length of heroin use, years	16.3 (±6.3)	10.5 (±5.0)	*t* = 6.9, *p* < .001
Cocktail use			
Moderate	20.3%	31.5%	*χ*^2^ = 4.5, *p* = .108
Intensive	70.3%	55.7%	
Multiple use of the same needle	91.9%	63.5%	*χ*^2^ = 20.1, *p* < .001
Needle/equipment sharing	93.2%	75.2%	*χ*^2^ = 10.6, *p* < .01

## Discussion and conclusion

This survey among 300 drug users of Kathmandu Valley is the first systematic study to shed a light on the current living situation, the state of health and drug use, as well as the risk behaviour of IDUs with concurrent consumption of opioids, benzodiazepines, antihistamines and/or other substances who are in contact with addiction services in Nepal. It provides current and specific knowledge on the status of HIV and other infections.

At present, the so-called South Asian cocktail, usually a combination of opioids, benzodiazepines and/or antihistamines, is the predominant drug in Nepal. The pharmaceutical drugs needed to prepare the mixture are less expensive than heroin and relatively easy to acquire. The drug users who consume opioids in combination with other medical substances have a higher risk behaviour than heroin drug users which is associated with the spread of HIV. It needs to be considered which HIV prevention measures are needed related to the specific needs of the so-called cocktail users, since the available services (such as needle syringe exchange) do not seem to cover their specific needs (high percentage of needle sharing). Respectively, it is necessary to identify why the available HIV prevention services (e.g. needle syringe exchange) do not cover the specific needs of drug addicts with combined drug use. Furthermore, OST services should be extended to decrease intravenous drug use effectively and to prevent infections of HIV, hepatitis and tuberculosis among IDUs. The level of OST coverage in Nepal is relatively low. As presented in the systematic review by Mathers et al. [15], the number of OST recipients in South Asia is 15 to 25 per 100 IDUs. Although the number of OST clinics increased from three to five in 2013 with a maximum number of 750 treatment slots, the coverage is still only about 2.5% of the intravenous drug users in Nepal.

With the normal pattern of consumption being to use these cocktails several times a day and the main reason for consumption being to alleviate psychological symptoms, this leads to the question whether users of drug mixtures practice a kind of self-medication of underlying mental symptoms or a kind of self-substitution of withdrawal symptoms, rather than using drugs in order to reach a ‘high’ or specific feelings. Especially diazepam has a long half-life period which produces a cumulative effect in the body. Thus, specific consequences and side effects of benzodiazepine dependence will arise after long-term concurrent use of opioids in combination with benzodiazepines and/or antihistamines. This indicates a much higher need for a comprehensive treatment approach in the region which provides the opportunity to offer appropriate treatment for other mental and somatic symptoms.

This study is the first of its kind to understand the situation of the so-called cocktail drug use in Nepal, and its results have shed some light on the circumstances of this special kind of combined drug use. At the same time, this research has detected the following more specific research needs: What is the specific situation of cocktail drug users with HIV infection? And how are other health needs of drug users who consume opioids in combination with benzodiazepines and/or antihistamines addressed, especially psychiatric co-morbidity? Furthermore, the specific role and effect (and possible risks) of the antihistamine within the mixture of substances has to be investigated.

Research also reveals that the substances for the drug mixture are catered by drug stores and chemists. This indicates a significant need for awareness raising among drugstore keepers, in order for them to understand the consequences of selling these drugs without a proper prescription.

With respect to harm reduction services and treatment offers, the question arises to what extent cocktail drug users utilize and benefit from harm reduction programmes. Furthermore, as buprenorphine is found to be the main substance involved in intravenous abuse of mixed drugs in Nepal, a need for scaling up buprenorphine substitution treatment within the OST program in Nepal has to be considered.

One limitation of our study is the choice of the sample which only consists of persons who are in contact with drug services or treatment. Thus, no information on drug users without professional support can be obtained, and we do not know how much we can extend these findings to the other substance users in Nepal. This would be of particular interest, as the majority of drug users in Nepal are not reached by special services. Thus, a national level survey is needed to understand the situation of drug users who consume opioids in combination with benzodiazepines and/or antihistamines in Nepal.

However, the specific problems of persons with concurrent use of opioids in combination with benzodiazepines, antihistamines and/or other substances should be covered by professional services which, again, require an increase in capacity of OST and harm reduction activities for drug users.

## Competing interests

The authors declare that they have no competing interests.

## Authors’ contributions

SPO, SS, H-GM-T and UV were involved in the study concept and design. SS carried out the data collection. SS, SPO and UV performed the statistical analyses and interpretation of data. SPO, SS, H-GM-T, HO and UV participated in the writing of the manuscript. All authors read and approved the final manuscript.
